# Mortality From Chronic Chagas Cardiomyopathy in Brazil: National and Regional Trends, 2000-2023

**DOI:** 10.7759/cureus.117681

**Published:** 2026-10-09

**Authors:** Humberto G Moreira, Beatriz Modesto P Reis, Lisia Raquel F Paz, Sofia de Souza F e Silva, Letícia C Tavares

**Affiliations:** 1 School of Medicine, Federal University of Goiás, Goiânia, BRA; 2 School of Medicine, Universidade de Uberaba, Uberaba, BRA; 3 School of Medicine, Centro Universitário UNINOVAFAPI, Teresina, BRA; 4 School of Medicine, Faculdade da Saúde e Ecologia Humana, Vespasiano, BRA; 5 School of Medicine, Faculdade de Medicina de Barbacena, Barbacena, BRA

**Keywords:** age-standardized rates, brazil, chagas cardiomyopathy, chagas disease, epidemiological trends, joinpoint regression, mortality, neglected tropical diseases, vital statistics

## Abstract

Background: Chronic Chagas cardiomyopathy is the main cause of death from *Trypanosoma cruzi* infection. Nationwide analyses of its mortality in Brazil are either restricted to all forms of Chagas disease or end before 2020, and none has examined the cardiac form separately across regions, sexes, and age groups through 2023.

Methods: This is a nationwide ecological time-trend study of all deaths registered in the Brazilian Mortality Information System with chronic Chagas disease with heart involvement (ICD-10 B57.2) as the underlying cause, 2000-2023. Age-standardized mortality rates (ASMR, direct method, Brazilian 2022 population as standard) were calculated for Brazil, its five macroregions, and each sex. Trends were estimated as annual percent change (APC) from age-adjusted quasi-Poisson regression and by joinpoint regression of the annual ASMR series, with the average APC (AAPC) for 2000-2023; sensitivity analyses used the World Health Organization (WHO) standard population and restricted windows (2006-2023; 2000-2019).

Results: Eighty-nine thousand seventy-nine deaths were attributed to B57.2 in 2000-2023 (56.1% male; 68.3% aged ≥60 years). The national ASMR fell from 4.06 (95% confidence interval (CI) 3.94-4.18) to 1.29 (1.24-1.34) per 100,000 population between 2000 and 2023 (−68%), with an AAPC of −4.7% (95% CI −4.9 to −4.5) and a single joinpoint in 2020, after which the decline accelerated (APC −6.9%, −8.7 to −5.2). Rates fell in all macroregions (APC from −2.6% in the Northeast to −6.5% in the South) and in both sexes (men −5.1%, women −3.9%), yet the Central-West retained an ASMR 3.5 times the national value in 2020-2023 (5.18 vs 1.49 per 100,000). The decline was steepest below 40 years of age (APC −9.9%) and negligible at ≥80 years (−0.5%, −0.7 to −0.2), in whom the rate remained above 20 per 100,000.

Conclusions: Mortality attributed to chronic Chagas cardiomyopathy in Brazil has declined by about two-thirds since 2000, in every region and both sexes. The age pattern is consistent with the aging and extinction of cohorts infected before vector control rather than with improved survival of established cardiomyopathy, although this ecological design cannot separate age, period, and cohort effects; deaths are now concentrated in elderly residents of historically endemic areas, particularly the Central-West.

## Introduction

Chagas disease remains the leading parasitic cause of heart disease in Latin America. The Global Burden of Disease (GBD) Study 2023 estimated 10.5 million prevalent infections and 8,420 deaths worldwide in 2023, with the highest age-standardized mortality in tropical Latin America, a region dominated by Brazil [1]. Chronic Chagas cardiomyopathy, which develops in 20% to 30% of infected people after a latency of two to three decades, accounts for most of this mortality through heart failure, ventricular arrhythmia, sudden death, and thromboembolism [2]. Once cardiomyopathy is established, the prognosis is poor: a meta-analysis of 52 cohorts reported an annual all-cause mortality of 7.9% (95% confidence interval (CI) 6.3-10.1), rising above 20% per year in advanced heart failure stages [3], and the worldwide prevalence of the cardiac form has fallen far more slowly than that of infection itself [4].

Brazil holds the largest absolute burden of the disease and the longest continuous record of cause-specific mortality in the region. Nationwide analyses of death certificates have documented a steady decline in Chagas-related mortality since the 1980s, alongside a persistent concentration of deaths in the Central-West and Southeast, in men, and at older ages [5-7]. The most recent national series, covering 2000-2019, reported an average annual decline of 3.1% in age-adjusted mortality from all forms of Chagas disease listed as underlying or associated cause [7], and a parallel analysis of hospital and death records found a much smaller decline (−0.7% per year) with an increase in the Northeast when associated causes were included [8]. These estimates are sensitive to coding: about one quarter of Chagas deaths may be hidden in ill-defined cardiac codes, and the proportion recovered by reclassification is highest in the most endemic states [9]. Burden-of-disease modeling for Brazil confirms that the decline is driven by premature mortality and that the residual burden sits in the states of historical vector transmission [10,11]. State-level studies from the Northeast, however, describe stable or even rising mortality in Sergipe, Bahia, Pernambuco, and Ceará over the last two decades [12-16].

Three gaps remain. First, the published national series either combine all clinical forms of Chagas disease [5,7,8] or end in 2014-2019 [6,7], so the trajectory of the cardiac form itself, the form that carries almost all of the mortality, is not described through the COVID-19 pandemic years. Second, where the cardiac form was examined nationally, it was reported as a proportion of Chagas deaths rather than as a rate with its own age-standardized trend [5,7]. Third, the apparent conflict between declining national rates and stable or rising rates in Northeastern states has not been examined with region-specific trend models and a common standard population.

We therefore analyzed all deaths attributed to chronic Chagas cardiomyopathy in Brazil from 2000 to 2023, using individual-level death records to isolate ICD-10 B57.2, to estimate age-standardized mortality trends for the country, its five macroregions, each sex, and each age group. We hypothesized that mortality would decline nationally, that the decline would be fastest in younger age groups, consistent with a birth-cohort effect from interrupted transmission, and that regional heterogeneity would persist.

## Materials and methods

Study design and data sources

This is a nationwide ecological study of time trends in routinely collected mortality data from the national vital registration system, with denominators from official population projections, reported according to the STROBE recommendations for observational studies [17]. Death records were obtained from the Mortality Information System (Sistema de Informações sobre Mortalidade, SIM) of the Brazilian Ministry of Health, which compiles all death certificates issued in the country and codes the underlying cause according to ICD-10 rules [18]. Individual-level, anonymized death records (the annual "DO" files for each of the 27 federative units, 2000-2023, 648 files in total) were downloaded from the DATASUS file transfer server and processed in Python (Python Software Foundation, Fredericksburg, VA, USA), because the public online tabulator (TabNet) reports causes only at the three-character ICD-10 level (B57) and cannot isolate the cardiac subcategory. Population denominators by federative unit, sex, and five-year age band were obtained from the Brazilian Institute of Geography and Statistics (IBGE) population projections, 2024 revision, which incorporate the 2022 Census and cover 2000 onward [19].

Case definition and study population

We included every death registered in SIM from January 1, 2000, to December 31, 2023, in which the underlying cause (field CAUSABAS) was coded B57.2, "Chagas disease (chronic) with heart involvement". The series begins in 2000, the first year for which the IBGE projections provide denominators by age, sex, and federative unit. Deaths were classified by year of death, sex, age at death, and macroregion of residence (North, Northeast, Southeast, South, and Central-West), the latter derived from the first digit of the IBGE municipality code of residence. Deaths in which B57.2 appeared only as an associated (contributory) cause were not included, so that the outcome represents deaths certified as directly due to chronic Chagas heart disease.

Of 89,079 eligible deaths, six (0.01%) had sex not stated and 28 (0.03%) had age not stated. Deaths with unknown sex contribute to all-sex analyses only, and deaths with unknown age contribute to counts and crude rates but not to age-specific or age-standardized rates; no imputation was performed. No death had missing region of residence.

Outcomes

The primary outcome was the annual age-standardized mortality rate (ASMR) per 100,000 population for Brazil and its temporal trend, expressed as the annual percent change (APC) and the average APC (AAPC) for 2000-2023. Secondary outcomes were the ASMR and its trend for each macroregion and each sex, the age-group-specific mortality rates and their trends, and the age-specific mortality pattern pooled over the study period.

Statistical analysis

Age at death was grouped into 17 bands (0-4, 5-9, …, 75-79, ≥80 years); the IBGE bands 80-84, 85-89, and ≥90 years were pooled to match the open upper band of the mortality data. Crude rates were computed as deaths divided by mid-year population. ASMRs were computed by the direct method using the 2022 Brazilian population, both sexes, as the standard (Appendix A), in accordance with the reference population adopted across our research program; 95% CIs were derived from the variance of the weighted sum of age-specific rates under the Poisson assumption. Rates were also pooled over five periods (2000-2004, 2005-2009, 2010-2014, 2015-2019, and 2020-2023) by summing deaths and person-years within age bands. As a sensitivity analysis, ASMRs were recomputed with the World Health Organization (WHO) World Standard Population [20].

Two complementary approaches were used to estimate trends. First, for each series (Brazil, each macroregion, each sex, and each of four broad age groups: <40, 40-59, 60-79, and ≥80 years), we fitted a Poisson regression of annual age-band-specific death counts with fixed effects for age band, a linear term for calendar year, and the logarithm of the age-band-specific population as offset; the APC was calculated as (eβ − 1) × 100, where β is the coefficient for year. Because the Pearson dispersion statistic exceeded 1 in all series (range 1.1-9.8; Appendix B), standard errors were multiplied by the square root of the dispersion (quasi-Poisson), and all CIs and P values for APCs are reported on this basis. Second, to identify changes in trend, we applied joinpoint regression to the annual ASMR series: the natural logarithm of the rate was regressed on calendar year with piecewise-linear segments, weighted by the inverse variance of the log rate, following the method of Kim et al. [21]. The number (0 to 3) and location of joinpoints were selected by the Bayesian information criterion (BIC) [21,22]. For each number of joinpoints k, every admissible set of locations on the observed years was fitted, with at least three observations between a joinpoint and either end of the series and at least four between joinpoints, and the set with the smallest weighted residual sum of squares (SSE_k) was retained; the model minimizing BIC_k = ln(SSE_k/n) + (2k + 2)·ln(n)/n was then selected, where n = 24 and 2k + 2 is the number of regression parameters. The BIC is one of the selection methods implemented in the Joinpoint software (National Cancer Institute, Bethesda, MD, USA); unlike the permutation test, it is deterministic and involves no resampling, and in short series, it tends to retain slightly more joinpoints [22]. Residual variance was estimated from the selected fit, and CIs used the t distribution. The AAPC for 2000-2023 was calculated as the length-weighted geometric mean of segment APCs, with CIs by the delta method. Joinpoint models were fitted for Brazil, each macroregion, and each sex.

Two-sided P values below 0.05 were considered statistically significant; no adjustment was made for multiplicity, and interpretation relies on effect sizes and CIs rather than on P values alone. As sensitivity analyses of the trend estimates, all models were refitted on two restricted windows: 2006-2023, which excludes the years in which ill-defined causes of death were most frequent in SIM and in which reclassification of such codes to Chagas disease is known to be largest [9], and 2000-2019, which excludes the COVID-19 pandemic years (Appendices C, D). Analyses were performed in Python 3.11 (NumPy 2.4, SciPy 1.17, pandas 3.0; Python Software Foundation, Fredericksburg, VA, USA), with the Poisson and joinpoint models implemented from their published specifications. Analysis code and the derived datasets are available from the authors.

Ethics

The study used publicly available, aggregated, and anonymized secondary data; under Brazilian National Health Council Resolution 510/2016, it does not require ethics committee review.

## Results

Deaths attributed to chronic Chagas cardiomyopathy

Between 2000 and 2023, 89,079 deaths in Brazil were attributed to chronic Chagas cardiomyopathy as the underlying cause (Table 1). Men accounted for 50,013 deaths (56.1%). More than two-thirds of decedents (68.3%) were 60 years or older, 27.2% were aged 40-59 years, and only 4.4% were younger than 40; the approximate mean age at death was 65.8 years. Half of all deaths (49.9%) occurred among residents of the Southeast, followed by the Central-West (23.0%), Northeast (21.1%), South (4.5%), and North (1.5%). Annual deaths decreased from 4,474 in 2000 to 2,810 in 2023 (Table 2).

**Table 1:** Characteristics of deaths from chronic Chagas cardiomyopathy (ICD-10 B57.2, underlying cause), Brazil, 2000-2023. Percentages calculated over the total number of deaths (N = 89,079) for the study period (2000-2023) and may not sum to 100.0% due to rounding. *Mean age at death estimated from the midpoint of each 5-year age band (0.5 years for <1 year, 2.5 years for 1-4 years, and 85 years for the open-ended ≥80 category); excludes deaths with age not stated (N = 28) [18]. ICD-10: International Classification of Diseases, 10th revision

Characteristic	N	%
Total deaths, N	89,079	100.0
Sex		
Male	50,013	56.1
Female	39,060	43.8
Sex not stated	6	0.0
Age group (years)		
0-19	120	0.1
20-39	3,807	4.3
40-59	24,247	27.2
60-79	44,706	50.2
≥80	16,171	18.2
Age not stated	28	0.0
Mean age at death (years) (approximate)*	65.8	-
Macroregion of residence		
Southeast	44,451	49.9
Northeast	18,777	21.1
Central-West	20,505	23.0
South	4,020	4.5
North	1,326	1.5

**Table 2:** Annual number of deaths, crude mortality rate, and age-standardized mortality rate from chronic Chagas cardiomyopathy (ICD-10 B57.2, underlying cause) in Brazil, 2000-2023. CI: confidence interval; ICD-10: International Classification of Diseases, 10th revision. Deaths: all deaths with ICD-10 B57.2 as the underlying cause registered in the Mortality Information System (SIM/DATASUS) [18], by year of death and place of residence. Population: mid-year resident population of Brazil, all ages, from the Brazilian Institute of Geography and Statistics (IBGE) 2024-edition population projections [19]. Crude rate = deaths ÷ population × 100,000. Age-standardized rate: direct method, standard population = Brazilian population in 2022 by 5-year age band (Appendix A); deaths with unknown age (n = 28 over the study period) are included in the death counts and crude rates but excluded from the age-standardized rates.

Year	Deaths, n	Population	Crude mortality rate (per 100,000 population)	Age-standardized mortality rate (per 100,000 population; 95% CI)
2000	4,474	174,695,935	2.56	4.06 (3.94-4.18)
2001	4,269	177,003,743	2.41	3.76 (3.64-3.87)
2002	4,212	179,228,254	2.35	3.61 (3.50-3.72)
2003	4,233	181,377,654	2.33	3.54 (3.43-3.65)
2004	4,284	183,469,593	2.33	3.49 (3.38-3.59)
2005	4,209	185,518,369	2.27	3.32 (3.22-3.42)
2006	4,019	187,535,030	2.14	3.08 (2.98-3.17)
2007	3,773	189,458,827	1.99	2.80 (2.71-2.89)
2008	4,065	191,335,196	2.12	2.93 (2.84-3.03)
2009	3,745	193,174,036	1.94	2.63 (2.55-2.72)
2010	3,805	194,749,329	1.95	2.60 (2.51-2.68)
2011	3,637	196,158,610	1.85	2.41 (2.33-2.49)
2012	3,646	197,670,620	1.84	2.35 (2.27-2.42)
2013	3,580	199,226,702	1.8	2.23 (2.16-2.31)
2014	3,436	200,811,131	1.71	2.08 (2.01-2.15)
2015	3,555	202,403,642	1.76	2.09 (2.02-2.15)
2016	3,561	203,871,925	1.75	2.02 (1.96-2.09)
2017	3,460	205,211,557	1.69	1.91 (1.84-1.97)
2018	3,372	206,529,038	1.63	1.80 (1.74-1.86)
2019	3,359	207,900,099	1.62	1.73 (1.67-1.79)
2020	3,265	209,164,889	1.56	1.63 (1.58-1.69)
2021	3,180	210,103,642	1.51	1.55 (1.49-1.60)
2022	3,130	210,862,983	1.48	1.48 (1.43-1.54)
2023	2,810	211,695,158	1.33	1.29 (1.24-1.34)

National decline in age-standardized mortality (primary outcome)

The crude mortality rate fell from 2.56 to 1.33 per 100,000 population between 2000 and 2023, and the ASMR from 4.06 (95% CI 3.94-4.18) to 1.29 (1.24-1.34) per 100,000, a relative reduction of 68% (Figure 1, Table 2). The age-adjusted quasi-Poisson model estimated an APC of −4.6% per year (95% CI −4.9 to −4.3; Table 3). Joinpoint regression identified one change in trend, in 2020: the ASMR declined by 4.4% per year (95% CI −4.5 to −4.2) in 2000-2020 and by 6.9% per year (−8.7 to −5.2) in 2020-2023, giving an AAPC of −4.7% (−4.9 to −4.5) for the whole period. The permutation procedure selected the same model (one joinpoint in 2020; P = 0.046 for one versus no joinpoint; Appendix E). Under the WHO World Standard, the ASMR fell from 3.33 to 1.03 per 100,000 (−69%), and regional rankings were unchanged (Appendix F).

**Figure 1:**
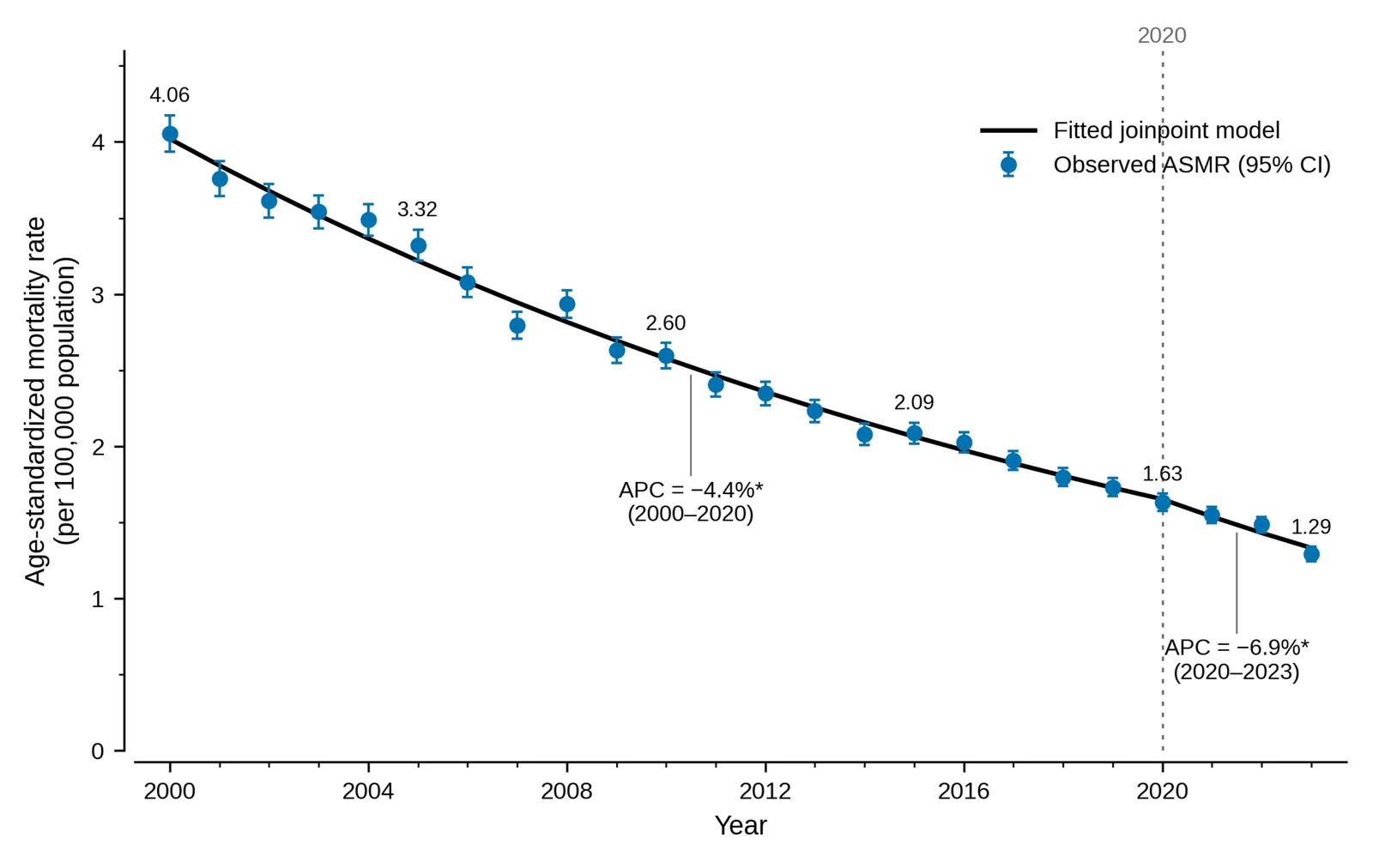
Temporal trend in age-standardized mortality from chronic Chagas cardiomyopathy in Brazil, 2000-2023. Points represent the observed annual ASMR per 100,000 population for deaths with chronic Chagas disease with heart involvement (ICD-10 B57.2) as the underlying cause, with 95% CIs (error bars). The solid line represents the fitted joinpoint regression model (weighted log-linear regression of the annual rate on calendar year, with joinpoints selected by the Bayesian information criterion); the vertical dotted line indicates the single joinpoint identified (2020). The APC of each segment is shown with its period: −4.4% per year (95% CI −4.5 to −4.2) for 2000-2020 and −6.9% per year (95% CI −8.7 to −5.2) for 2020-2023. Rates were directly standardized to the 2022 Brazilian population (Appendix A). Average APC for the whole period (2000-2023): −4.7% (95% CI −4.9 to −4.5). Source: Mortality Information System (SIM/DATASUS), Brazilian Ministry of Health [18]; population denominators from the Brazilian Institute of Geography and Statistics (IBGE) [19]. *APC significantly different from zero (P < 0.05). APC: annual percent change; ASMR: age-standardized mortality rate; CI: confidence interval; ICD-10: International Classification of Diseases, 10th revision

**Table 3:** Temporal trend in age-standardized mortality from chronic Chagas cardiomyopathy, Brazil, 2000-2023: age-adjusted Poisson regression and joinpoint regression. *APC = (e^β^ − 1) × 100, where β is the coefficient for calendar year (continuous) in a Poisson regression of annual age-band-specific death counts with fixed effects for 5-year age band and the logarithm of the age-band-specific population as offset. Pearson dispersion statistics ranged from 1.1 (North) to 9.8 (Brazil overall) (Appendix B); CIs and P values were therefore obtained from quasi-Poisson models with standard errors scaled by the square root of the dispersion. ^†^Joinpoint regression of the natural logarithm of the annual age-standardized rate on calendar year, weighted by the inverse variance of the log rate, with the number and location of joinpoints (0 to 3) selected by the Bayesian information criterion under the constraints of at least three observations between a joinpoint and either end of the series and at least four observations between joinpoints; one joinpoint was identified (2020). AAPC is the length-weighted geometric mean of segment-specific APCs; its CI was obtained by the delta method. Region- and sex-specific joinpoint models are shown in Appendix G. APC: annual percent change; AAPC: average annual percent change; CI: confidence interval

Series/period	APC, % per year (95% CI)	P value
Overall trend, 2000-2023 (age-adjusted Poisson regression)*		
Brazil (overall)	−4.6 (−4.9 to −4.3)	<0.001
North	−3.4 (−4.2 to −2.6)	<0.001
Northeast	−2.6 (−3.0 to −2.3)	<0.001
Southeast	−5.3 (−5.6 to −5.0)	<0.001
South	−6.5 (−7.0 to −6.0)	<0.001
Central-West	−5.4 (−5.7 to −5.0)	<0.001
Male (Brazil)	−5.1 (−5.4 to −4.8)	<0.001
Female (Brazil)	−3.9 (−4.3 to −3.6)	<0.001
Joinpoint regression, Brazil (overall)^†^		
2000-2020	−4.4 (−4.5 to −4.2)	<0.001
2020-2023	−6.9 (−8.7 to −5.2)	<0.001
AAPC, 2000-2023	−4.7 (−4.9 to −4.5)	<0.001

Regional trends

All five macroregions showed statistically significant declines, but their magnitude and shape differed (Figure 2, Tables 3, 4, Appendix G). The Central-West had by far the highest mortality throughout: its period ASMR fell from 15.30 (95% CI 14.87-15.73) per 100,000 in 2000-2004 to 5.18 (4.99-5.37) in 2020-2023, i.e., from 4.2 to 3.5 times the national rate and 3.6 times the Southeast rate in the first period. Its trend had two joinpoints, with rapid decline in 2000-2007 (APC −7.5%), a slower phase in 2007-2018 (−3.1%), and renewed acceleration after 2018 (−9.1%). The Southeast and South declined steadily without joinpoints (joinpoint APC −5.2% and −6.3% per year); the South reached the lowest rate of any region by 2023 (0.30 per 100,000). In contrast, the Northeast showed no decline until 2008 (APC +0.7%, 95% CI −0.3 to 1.8) and a moderate decline thereafter (−3.8%, −4.3 to −3.3), the smallest overall change of any region (AAPC −2.2%). The North had the lowest and least precise rates (0.73 in 2000, 0.31 in 2023, with wide CIs reflecting fewer than 100 deaths per year), with no significant change until 2016 and a steep decline afterward (−9.8% per year, −12.2 to −7.4). The permutation procedure selected the same joinpoint model as the BIC in all five macroregions (Appendix E).

**Figure 2:**
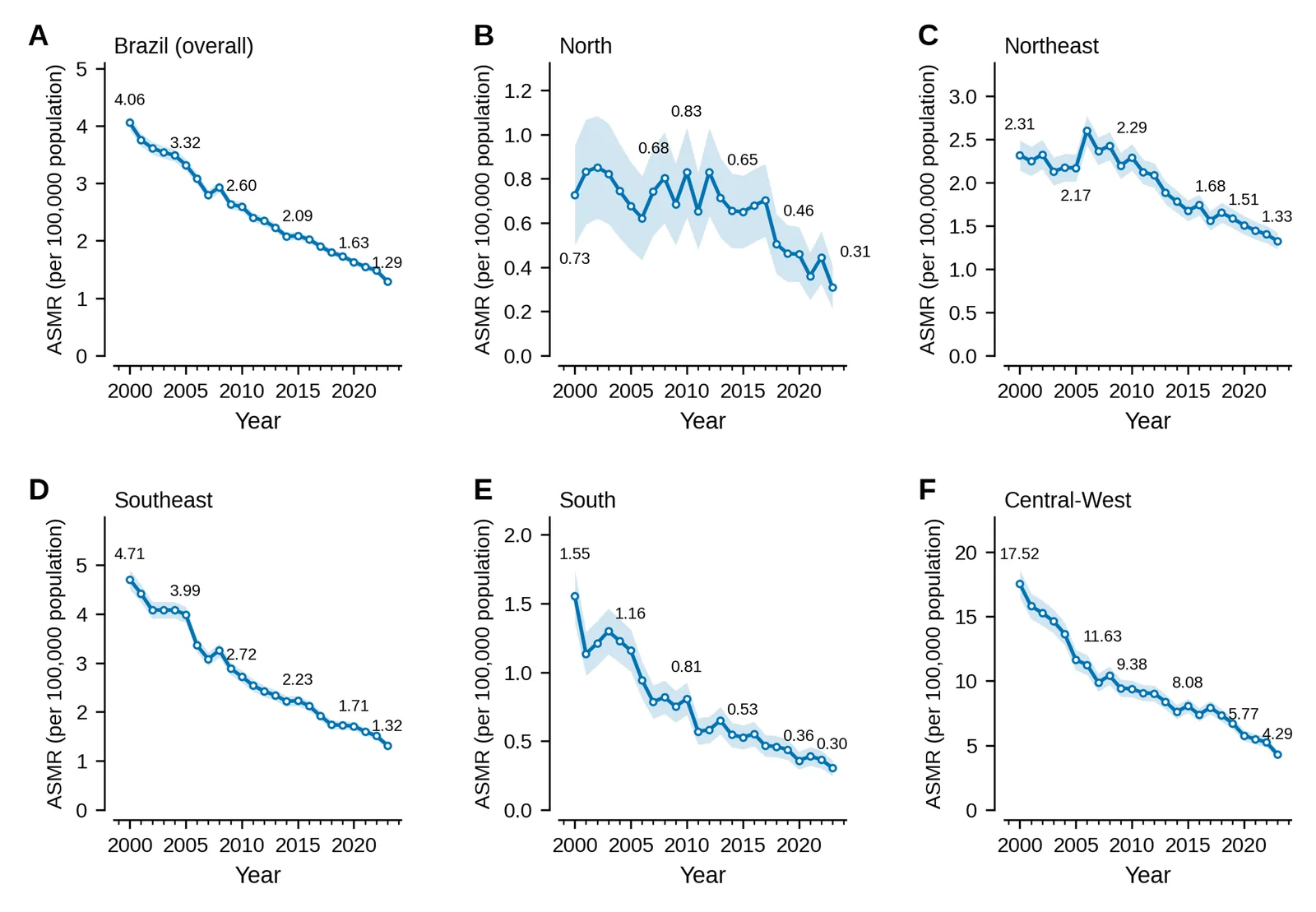
Age-standardized mortality from chronic Chagas cardiomyopathy in Brazil (A) and in each of its five macroregions (B-F), 2000-2023. (A) Brazil, overall; (B) North; (C) Northeast; (D) Southeast; (E) South; (F) Central-West. Lines with open circles represent the annual ASMR per 100,000 population for ICD-10 B57.2 as the underlying cause of death; shaded bands represent 95% confidence intervals. Rates were directly standardized to the 2022 Brazilian population (Appendix A). Note that the y-axis scale differs between panels to accommodate the wide between-region differences in mortality level; the Central-West (F) shows rates several-fold higher than the other regions throughout the period. Source: Mortality Information System (SIM/DATASUS) [18]; population denominators from Brazilian Institute of Geography and Statistics (IBGE) [19]. ASMR: age-standardized mortality rate; ICD-10: International Classification of Diseases, 10th revision

**Table 4:** Age-standardized mortality rate (per 100,000 population) from chronic Chagas cardiomyopathy, by 5-year period and macroregion, Brazil, 2000-2023. Values are age-standardized mortality rates per 100,000 population (95% confidence interval), computed by the direct method using the 2022 age structure of the Brazilian population (Brazilian Institute of Geography and Statistics (IBGE) 2024-edition population projections) as the standard population, pooled across each 5-year period [19].

Macroregion	2000-2004	2005-2009	2010-2014	2015-2019	2020-2023
Brazil (overall)	3.68 (3.63-3.73)	2.94 (2.90-2.98)	2.32 (2.29-2.36)	1.90 (1.87-1.93)	1.49 (1.46-1.51)
North	0.80 (0.69-0.90)	0.71 (0.62-0.80)	0.73 (0.65-0.82)	0.59 (0.53-0.66)	0.39 (0.34-0.45)
Northeast	2.23 (2.16-2.31)	2.35 (2.28-2.42)	2.02 (1.96-2.09)	1.64 (1.59-1.69)	1.42 (1.37-1.47)
Southeast	4.26 (4.19-4.34)	3.30 (3.24-3.37)	2.44 (2.39-2.49)	1.94 (1.90-1.98)	1.53 (1.49-1.57)
South	1.28 (1.21-1.36)	0.89 (0.83-0.94)	0.63 (0.58-0.67)	0.49 (0.45-0.52)	0.35 (0.32-0.39)
Central-West	15.30 (14.87-15.73)	10.48 (10.16-10.81)	8.66 (8.39-8.93)	7.47 (7.25-7.70)	5.18 (4.99-5.37)

Sex-specific trends

Men had higher mortality than women in every year. The male ASMR fell from 5.13 (95% CI 4.93-5.33) to 1.54 (1.46-1.61) per 100,000, and the female ASMR from 3.11 (2.97-3.25) to 1.09 (1.03-1.15), so the male-to-female ratio narrowed from 1.65 to 1.41 (Figure 3). The age-adjusted APC was −5.1% per year (95% CI −5.4 to −4.8) in men and −3.9% (−4.3 to −3.6) in women (Table 3). Joinpoint models placed the acceleration of decline in 2019 for men (APC −6.9% thereafter) and 2020 for women (−6.2%) (Appendix G). These sex-specific joinpoints were not retained by the permutation procedure (P = 0.063 in men and 0.079 in women; Appendix E), so the timing of the acceleration within each sex should be regarded as uncertain.

**Figure 3:**
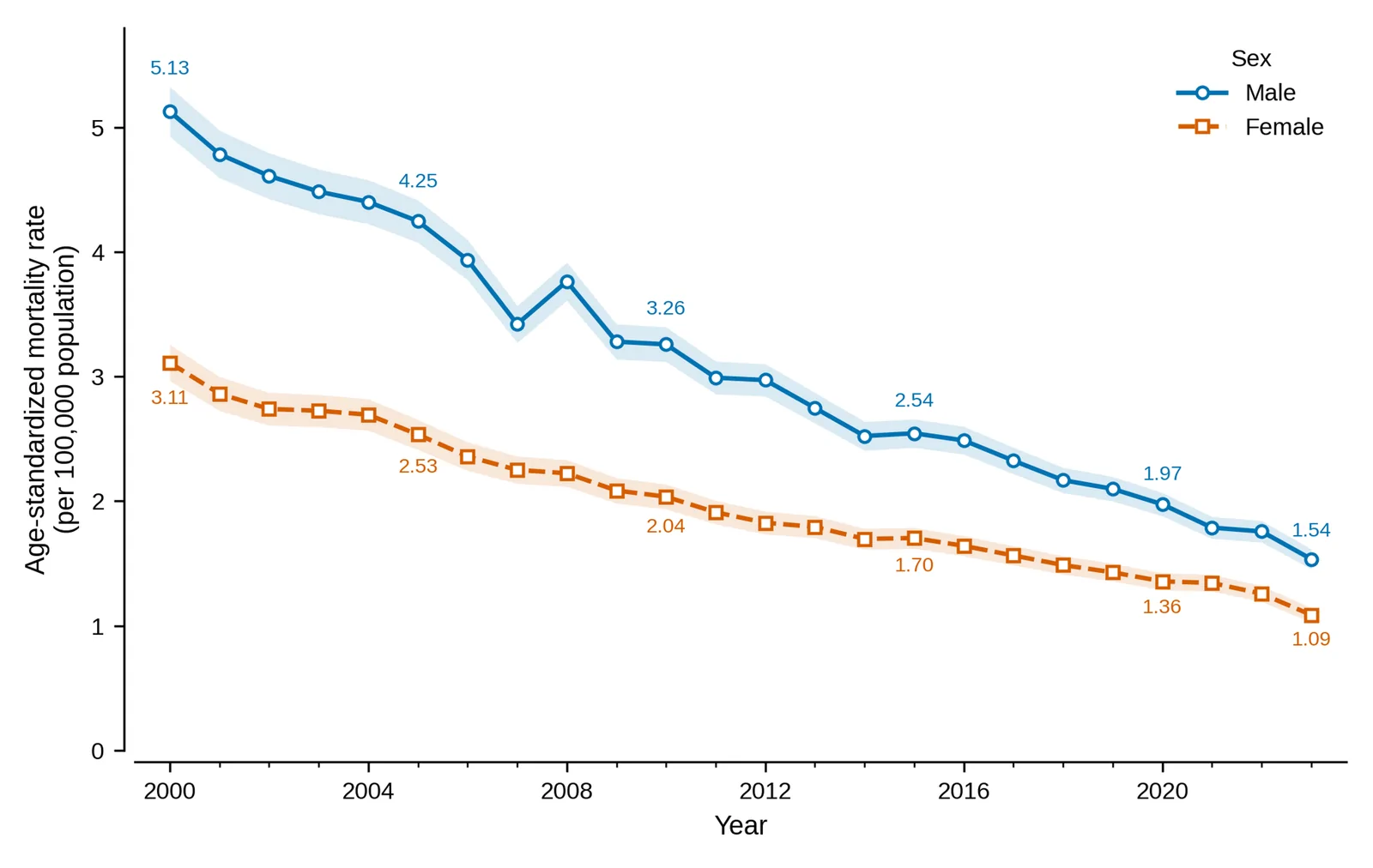
Sex-specific trends in age-standardized mortality from chronic Chagas cardiomyopathy in Brazil, 2000-2023. Age-standardized mortality rate per 100,000 population for ICD-10 B57.2 as the underlying cause of death, by sex: males (solid line, open circles) and females (dashed line, open squares). Shaded bands represent 95% CIs. Rates were directly standardized to the 2022 Brazilian population (Appendix A). Age-adjusted annual percent change from quasi-Poisson regression: males −5.1% per year (95% CI −5.4 to −4.8); females −3.9% per year (95% CI −4.3 to −3.6); both P < 0.001 (Table 3). Source: Mortality Information System (SIM/DATASUS) [18]; population denominators from Brazilian Institute of Geography and Statistics (IBGE) [19]. CI: confidence interval; ICD-10: International Classification of Diseases, 10th revision

Age pattern

Mortality rose steeply with age: pooled over 2000-2023, the rate was below 0.05 per 100,000 before 20 years, about one per 100,000 at 40-44 years, and above 20 per 100,000 at 80 years or older (Figure 4). The trend was strongly age-dependent (Appendix H). Between 2000-2004 and 2019-2023, the rate fell by 85% below 40 years of age (APC −9.9% per year, 95% CI −10.5 to −9.3), by 75% at 40-59 years (−7.3%, −7.5 to −7.1), and by 57% at 60-79 years (−4.2%, −4.5 to −3.9), but by only 10% at 80 years or older (22.3 to 20.1 per 100,000; APC −0.5%, −0.7 to −0.2). This heterogeneity across age groups accounts for most of the extra-Poisson variation detected in the age-adjusted models (Appendix B).

**Figure 4:**
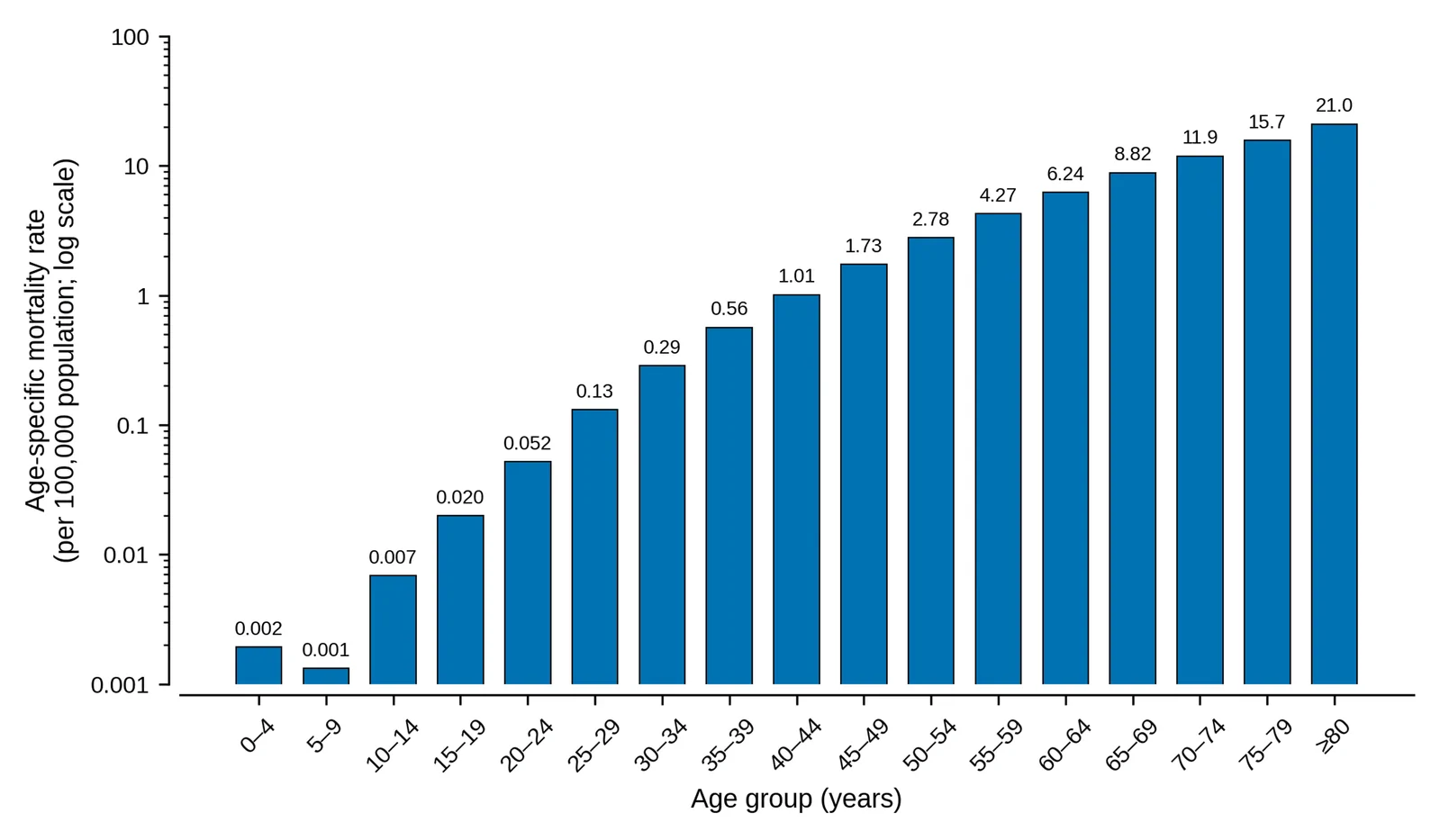
Age-specific mortality from chronic Chagas cardiomyopathy in Brazil, 2000-2023 (pooled). Bars represent the age-specific mortality rate per 100,000 population (logarithmic scale) for ICD-10 B57.2 as the underlying cause of death, by 5-year age band, calculated as the total number of deaths in each age band divided by the corresponding total person-years over 2000-2023. Deaths with unknown age (n = 28 over 2000-2023) were excluded. Mortality rises steeply from the fifth decade of life onward, consistent with the latency of two to three decades between *Trypanosoma cruzi* infection and clinically manifest cardiomyopathy. Source: Mortality Information System (SIM/DATASUS) [18]; population denominators from Brazilian Institute of Geography and Statistics (IBGE) [19]. ICD-10: International Classification of Diseases, 10th revision

Sensitivity analyses

Restricting the series to 2006-2023 left the national APC unchanged (−4.6% per year, 95% CI −5.0 to −4.2), while the declines in the Northeast (−3.8%) and North (−4.6%) became steeper, consistent with their later onset (Appendix C); the 2020 joinpoint was preserved in this window (Appendix D). Restricting the series to 2000-2019 gave a national APC of −4.5% (−4.8 to −4.2) and no joinpoint (Appendices C, D), indicating that the change in slope identified in the main analysis is confined to the 2020-2023 observations.

## Discussion

In this nationwide analysis of 24 years of individual death records, mortality attributed to chronic Chagas cardiomyopathy in Brazil fell by about two-thirds, at an average of 4.7% per year, in every macroregion and in both sexes. The decline itself is not a new observation; every national series since the 1990s has reported it for Chagas disease as a whole [5-8]. What this study adds is its resolution: the trend of the cardiac form specifically, isolated from other clinical forms, followed through 2023 and broken down by region, sex, and age. Three features of the decline are informative. It was steepest in adults under 40 and progressively weaker with age, to the point of being almost absent above 80 years. It accelerated after 2020. And it did not erase regional inequality: the Central-West still had an age-standardized rate three and a half times the national value in 2020-2023, and the Northeast, where the decline only began around 2008, has now overtaken the Southeast in relative terms.

The age gradient is the finding with the clearest mechanistic reading, although that reading is an inference rather than a demonstration. A decline that is concentrated in the young and nearly absent in the very old is the pattern expected under cohort replacement: successive birth cohorts carry a lower prevalence of *Trypanosoma cruzi* infection because they were born after domiciliary transmission by *Triatoma infestans* was interrupted, a process that began with the national vector-control program in 1975 and culminated in the certification of interruption of *T. infestans* transmission in the mid-2000s [23]. People now dying at 80 years or older were infected before 1960 and were fully exposed; their mortality is governed by the natural history of a cardiomyopathy acquired decades ago and by competing causes of death, not by current transmission. The same gradient was predicted by the Bayesian age-period-cohort model of Simões et al., who forecast the largest reductions in the youngest age groups and the smallest above 80 years [6], and it is consistent with the shift of the global age distribution of prevalent cases toward older ages in the GBD 2023 estimates [1]. If this interpretation is correct, the national decline should not be read as evidence that the case fatality of established cardiomyopathy has improved; the cohort data summarized by Chadalawada et al. show an annual mortality near 8% that has not changed materially over decades [3], and in the contemporary Brazilian BREATHE registry, patients hospitalized for acute heart failure due to Chagas cardiomyopathy had a 12-month mortality of 39.4%, about twice that of other etiologies [24]. What would have changed is the number of people reaching the age of cardiac expression with the infection. We stress that this reading is inferential: our data are period-specific rates by age group, not the follow-up of birth cohorts, and age, period, and cohort effects cannot be separated without additional assumptions. Secular changes in diagnosis, certification practice, competing causes of death, and access to care over 24 years could each contribute to a steeper decline in younger adults than in the very old, and cannot be excluded with these data.

Our national estimate of −4.7% per year is steeper than the −3.1% reported by Martins-Melo et al. for 2000-2019 [7] and much steeper than the −0.7% found by Souza et al. for the same years [8]. Both earlier studies counted all forms of Chagas disease and, decisively, included deaths in which the disease was an associated rather than the underlying cause. Associated-cause mentions have become more frequent as multiple-cause coding improved and as decedents with Chagas disease live long enough to die of other conditions, which dilutes the decline in underlying-cause mortality; the same mechanism explains why Souza et al. observed an increase in the Northeast where we observe a delayed decline [8]. Our restriction to B57.2 as underlying cause therefore isolates deaths certified as due to the cardiomyopathy, at the cost of excluding deaths in which the cardiomyopathy contributed without being selected as the underlying cause. The two designs answer different questions, and the difference between −4.7% and −0.7% is itself informative about how much of the residual burden is being recorded as a contributory condition. Reassuringly, the regional and sex patterns we describe match those of every previous national series: highest rates in the Central-West, male predominance, and older age at death [5-7].

The regional heterogeneity has two components. The absolute level of mortality mirrors the historical geography of vector transmission, with Goiás, Minas Gerais, and the Federal District long identified as the highest-risk states [5,10]. The timing of the decline, however, differs. The Southeast and South, which achieved vector control early and have the most complete death certification, show monotonic declines. The Central-West shows the fastest overall decline but with a decade-long deceleration (2007-2018) that we cannot explain with these data and that warrants examination at the state level. The Northeast is the outlier: no decline until 2008 and the smallest overall reduction. Three non-exclusive explanations deserve consideration. Transmission by *Triatoma brasiliensis* and *Triatoma pseudomaculata*, native species that recolonize dwellings, was interrupted later and less completely than *T. infestans* transmission, so Northeastern cohorts born in the 1960s-1980s may carry a higher residual prevalence [23]. Certification has improved: reclassification of ill-defined cardiac codes recovers proportionally more Chagas deaths in states of higher endemicity [9], and progressive improvement in cause-of-death investigation in SIM over the study period would raise apparent B57.2 mortality in regions with historically poorer certification before the true decline became visible. In addition, the state-level studies from Sergipe, Bahia, Pernambuco, and Ceará that report stable or rising mortality [12-16] describe exactly the period and region in which our national model places the plateau, so the apparent conflict between national and state analyses is one of window and of standard population rather than of substance. The very low rates in the North also deserve caution: in the Amazon region, where oral transmission predominates and specialized cardiology services are sparse, low recorded mortality from Chagas cardiomyopathy may reflect underdiagnosis as much as low occurrence [25].

The acceleration after 2020 coincides with the COVID-19 pandemic and should be interpreted with caution. Two mechanisms could produce an artefactual steepening. Excess mortality in 2020-2021 was large, unevenly recorded across regions and concentrated in older adults [26]; deaths of people with chagasic cardiomyopathy may have been certified as COVID-19, which would remove them from the B57.2 numerator without reducing the true burden of the disease; if so, the 2020-2023 segment partly reflects competing-cause displacement rather than a change in the underlying trend. Alternatively, mortality displacement may have "harvested" frail patients in 2020-2021, lowering counts in 2022-2023. A genuine acceleration is also plausible as the pre-control cohorts age out; the BIC-selected model for men placed a joinpoint in 2019, before the pandemic, but this joinpoint was not retained by the permutation procedure and should not be given weight. With only four post-joinpoint observations, the segment estimate is imprecise (95% CI −8.7 to −5.2), and when the series is truncated at 2019, no joinpoint is detected; we therefore regard the long-run decline of about 4.5% per year, rather than the post-2020 acceleration, as the robust finding.

The narrowing of the sex ratio, from 1.65 to 1.41, arises because men's mortality fell faster (−5.1% vs −3.9% per year). Male predominance in Chagas mortality has been attributed to greater occupational exposure, later diagnosis, and a higher incidence of ventricular arrhythmia and sudden death [2,3]. A faster decline in men is what one expects if the excess male mortality is concentrated in the cohort-driven component of the disease, but data cannot distinguish this from differential improvements in care.

Clinical and public-health implications

These results do not support a change in clinical management of individual patients; they describe who is dying of the disease. For health services, the message is that the population at risk of dying from chagasic cardiomyopathy is now old, predominantly male, and geographically concentrated. Cardiology and heart-failure services in the Central-West and in the interior of the Northeast will continue to see these patients for at least two more decades, and the near-absence of decline above 80 years means that the demand for care of advanced chagasic heart failure in the elderly will fall much more slowly than headline mortality. The recommendations of the 2023 Brazilian Society of Cardiology guideline on screening of at-risk individuals and on staged management of the cardiomyopathy [2] apply with particular force in these regions. For surveillance, the sensitivity of trend estimates to the choice between underlying and multiple causes, and to garbage-code reclassification, argues for reporting both in future national series.

Strengths and limitations

The strengths of the study are its complete national coverage, the use of individual death records to isolate the cardiac form, the 24-year window with consistent ICD-10 coding, the parallel use of two trend methods with explicit handling of overdispersion, and the sensitivity analysis with an international standard population.

The limitations are specific, and their likely direction can be stated. First, the outcome depends on death certification. Under-ascertainment of Chagas disease in ill-defined cardiac codes is substantial, larger in endemic states, and has decreased over time as certificate investigation improved [9]; this would understate absolute rates throughout and, by recovering more deaths in later years, bias the estimated decline toward the null, so the true decline may be somewhat steeper than reported; excluding 2000-2005, the period of poorest certification, did not change the national estimate. A quantitative correction was not attempted because the published reclassification factors refer to 2015-2019 and are not available by year, region, and age. Second, restriction to the underlying cause excludes deaths in which the cardiomyopathy contributed to death without being selected as its underlying cause; our rates therefore represent a lower bound of cardiomyopathy-related mortality, and the difference between our estimates and multiple-cause series [7,8] is expected. Third, the North region contributes small numbers (fewer than 100 deaths in most years), and its segment estimates should be regarded as imprecise; the same caution applies to the final 2020-2023 segment in every series. Fourth, the study is ecological and cannot examine individual-level determinants, race, socioeconomic status, or access to care, which previous work has shown to be associated with Chagas mortality [7,8,15]. Fifth, the cohort-replacement interpretation is inferential: the study analyzes period-specific mortality by age, and neither follows birth cohorts nor fits a formal age-period-cohort model, so the age gradient is consistent with, but does not establish, cohort replacement as the cause of the decline. Finally, no attempt was made to estimate excess or displaced mortality in 2020-2021, so the post-2020 acceleration remains provisional.

## Conclusions

Between 2000 and 2023, mortality certified as due to chronic Chagas cardiomyopathy in Brazil declined by roughly two-thirds, at an average of 4.7% per year, in all five macroregions and in both sexes, with an acceleration after 2020 that coincides with the pandemic and requires confirmation. The age-specific pattern, steepest in people under 40 and almost absent above 80, is consistent with a cohort phenomenon, and the decline has not removed the fourfold regional gradient that concentrates the disease in the Central-West. Chagas cardiomyopathy in Brazil is becoming a disease of elderly residents of formerly endemic areas, and health services and surveillance should be organized around that population.

## Appendices

Appendix A

**Table 5:** Standard population used for direct age standardization: resident population of Brazil in 2022 by 5-year age band, with the corresponding weights. Brazilian Institute of Geography and Statistics (IBGE), Projection of the population of Brazil and Federative Units by sex and age, 2000-2070 (2024 edition), both sexes, year 2022, summed over the 27 Federative Units. The open-ended band ≥80 years pools the IBGE bands 80-84, 85-89, and ≥90 years to match the upper age band available in the mortality data. Weights wᵢ = populationᵢ ÷ total population; the age-standardized rate for a given population and year was computed as Σᵢ (wᵢ × age-specific rateᵢ). This standard corresponds to the program-wide decision to use the IBGE 2022 Census-based population as the reference for direct standardization.

Age group (years)	Population, n	Weight (wᵢ)	Weight (%)
0-4	13,820,837	0.0655	6.55
5-9	14,588,148	0.0692	6.92
10-14	14,575,329	0.0691	6.91
15-19	15,402,409	0.0730	7.30
20-24	16,396,616	0.0778	7.78
25-29	16,468,095	0.0781	7.81
30-34	16,272,969	0.0772	7.72
35-39	16,815,501	0.0797	7.97
40-44	16,252,122	0.0771	7.71
45-49	14,076,325	0.0668	6.68
50-54	12,689,175	0.0602	6.02
55-59	11,640,958	0.0552	5.52
60-64	9,914,466	0.0470	4.70
65-69	7,821,332	0.0371	3.71
70-74	5,784,463	0.0274	2.74
75-79	3,903,952	0.0185	1.85
≥80	4,440,286	0.0211	2.11
Total	210,862,983	1.0000	100.00

Appendix B

**Table 6:** Overdispersion diagnostics of the age-adjusted Poisson trend models and comparison of Poisson and quasi-Poisson CIs. Dispersion = Pearson χ² divided by residual degrees of freedom for the model with age-band fixed effects, a linear year term, and log-population offset (408 age-band × year observations per series). Values above 1 indicate extra-Poisson variation, largely attributable to age-by-period interaction (heterogeneous trends across age groups, Appendix H). Quasi-Poisson CIs (standard errors multiplied by the square root of the dispersion) are reported in Table 3 and were used for inference throughout. APC: annual percent change; CI: confidence interval

Series	APC, % per year	Pearson dispersion (χ²/df)	95% CI, Poisson	95% CI, quasi-Poisson
Brazil (overall)	−4.60	9.80	−4.70 to −4.51	−4.89 to −4.32
North	−3.38	1.07	−4.13 to −2.63	−4.15 to −2.60
Northeast	−2.62	3.22	−2.82 to −2.42	−2.98 to −2.26
Southeast	−5.26	5.55	−5.39 to −5.13	−5.57 to −4.96
South	−6.52	1.27	−6.94 to −6.09	−7.00 to −6.04
Central-West	−5.36	3.19	−5.54 to −5.17	−5.69 to −5.02
Male (Brazil)	−5.09	5.41	−5.21 to −4.97	−5.38 to −4.81
Female (Brazil)	−3.94	5.00	−4.08 to −3.81	−4.25 to −3.64

Appendix C 

**Table 7:** Sensitivity of the age-adjusted annual percent change to the analytic window: main analysis (2000-2023) versus exclusion of the years of poorest cause-of-death certification (2006-2023) and of the COVID-19 pandemic years (2000-2019). Age-adjusted quasi-Poisson regression (same specification as Table 3) refitted within each analytic window. The 2006-2023 window excludes the years in which ill-defined causes of death were most frequent in the Mortality Information System; the 2000-2019 window excludes the COVID-19 pandemic years. All P values were < 0.001. APC: annual percent change; CI: confidence interval

Series	APC, % per year (95% CI) 2000-2023 (main)	APC, % per year (95% CI) 2006-2023 (garbage-code era excluded)	APC, % per year (95% CI) 2000-2019 (pre-pandemic)
Brazil (overall)	−4.6 (−4.9 to −4.3)	−4.6 (−5.0 to −4.2)	−4.5 (−4.8 to −4.2)
North	−3.4 (−4.2 to −2.6)	−4.6 (−5.7 to −3.5)	−2.2 (−3.2 to −1.2)
Northeast	−2.6 (−3.0 to −2.3)	−3.8 (−4.3 to −3.3)	−2.3 (−2.8 to −1.9)
Southeast	−5.3 (−5.6 to −5.0)	−5.1 (−5.5 to −4.6)	−5.3 (−5.7 to −5.0)
South	−6.5 (−7.0 to −6.0)	−5.9 (−6.6 to −5.2)	−6.6 (−7.2 to −6.0)
Central-West	−5.4 (−5.7 to −5.0)	−4.8 (−5.3 to −4.4)	−5.1 (−5.5 to −4.7)
Male (Brazil)	−5.1 (−5.4 to −4.8)	−5.2 (−5.6 to −4.8)	−5.0 (−5.3 to −4.6)
Female (Brazil)	−3.9 (−4.3 to −3.6)	−3.8 (−4.3 to −3.4)	−3.9 (−4.3 to −3.5)

Appendix D 

**Table 8:** Sensitivity of the national joinpoint model to the analytic window: main analysis (2000-2023) versus 2006-2023 and 2000-2019, Brazil, age-standardized mortality from chronic Chagas cardiomyopathy. Joinpoint regression of the national annual age-standardized rate within each window (same specification and selection rules as Table 3: weighted log-linear regression, Bayesian information criterion, 0 to 3 joinpoints). AAPC refers to the whole window. The 2006-2023 window excludes the years in which ill-defined causes of death were most frequent in the Mortality Information System; the 2000-2019 window excludes the COVID-19 pandemic years. APC: annual percent change; AAPC: average annual percent change; CI: confidence interval

Analytic window	Joinpoint(s)	Segment	APC, % per year (95% CI)	AAPC, % (95% CI)
2000-2023 (main)	2020	2000-2020	−4.4 (−4.5 to −4.2)	−4.7 (−4.9 to −4.5)
		2020-2023	−6.9 (−8.7 to −5.2)	
2006-2023 (garbage-code era excluded)	2020	2006-2020	−4.2 (−4.5 to −3.9)	−4.8 (−5.1 to −4.5)
		2020-2023	−7.1 (−9.0 to −5.3)	
2000-2019 (pre-pandemic)	None	2000-2019	−4.4 (−4.6 to −4.2)	−4.4 (−4.6 to −4.2)

Appendix E 

**Table 9:** Joinpoint model selection by the BIC (main analysis) and by the permutation procedure, for each series, Brazil, 2000-2023. Both procedures were applied to the same weighted log-linear piecewise regression of the annual age-standardized rate on calendar year (weights = inverse variance of the log rate; joinpoints at observed years; at least three observations between a joinpoint and either end of the series and at least four between joinpoints; 0 to 3 joinpoints). Permutation procedure of Kim et al. [21]: the null and alternative models are compared by the ratio of their weighted residual sums of squares; the reference distribution was obtained from 4,499 permutations of the weighted residuals of the null model; tests proceed sequentially from the maximum number of joinpoints downward, each at significance level α/(k_max − k_min) with overall α = 0.05 (Bonferroni adjustment); the first rejection raises the minimum number of joinpoints and a non-rejection lowers the maximum, until they coincide. P values are Monte Carlo estimates; across three additional random seeds, the P value for one versus no joinpoint ranged from 0.040 to 0.047 for Brazil, 0.053 to 0.063 for men, and 0.068 to 0.079 for women. The two procedures selected the same model in six of eight series; in the two sex-specific series, the BIC selected one joinpoint that the permutation procedure did not retain at the 5% level, consistent with the known tendency of the BIC to select more joinpoints in short series [22]. BIC: Bayesian information criterion

Series	BIC-selected model (joinpoint years)	Permutation-selected model (joinpoint years)	Sequence of permutation tests (H0 vs H1 joinpoints: P value; adjusted α)	Agreement
Brazil (overall)	1 (2020)	1 (2020)	0 vs 3: 0.124 (0.017); 0 vs 2: 0.134 (0.025); 0 vs 1: 0.046 (0.050)	Yes
North	1 (2016)	1 (2016)	0 vs 3: 0.001 (0.017); 1 vs 3: 0.576 (0.025); 1 vs 2: 0.733 (0.050)	Yes
Northeast	1 (2008)	1 (2008)	0 vs 3: <0.001 (0.017); 1 vs 3: 0.144 (0.025); 1 vs 2: 0.336 (0.050)	Yes
Southeast	0	0	0 vs 3: 0.266 (0.017); 0 vs 2: 0.383 (0.025); 0 vs 1: 0.692 (0.050)	Yes
South	0	0	0 vs 3: 0.474 (0.017); 0 vs 2: 0.483 (0.025); 0 vs 1: 0.623 (0.050)	Yes
Central-West	2 (2007, 2018)	2 (2007, 2018)	0 vs 3: <0.001 (0.017); 1 vs 3: <0.001 (0.025); 2 vs 3: 0.542 (0.050)	Yes
Male (Brazil)	1 (2019)	0	0 vs 3: 0.174 (0.017); 0 vs 2: 0.122 (0.025); 0 vs 1: 0.063 (0.050)	No
Female (Brazil)	1 (2020)	0	0 vs 3: 0.072 (0.017); 0 vs 2: 0.062 (0.025); 0 vs 1: 0.079 (0.050)	No

Appendix F

**Table 10:** Sensitivity of period age-standardized mortality rates to the choice of standard population: Brazilian 2022 population versus WHO World Standard Population. The WHO World Standard Population (2000-2025) weights were collapsed to the 17 age bands used in this study (≥80 years = 80-84 plus ≥85) [20]. Because the WHO standard is younger than the 2022 Brazilian population, absolute rates are lower under the WHO standard, but the ranking of regions and the relative decline between periods are unchanged. ASMR: age-standardized mortality rate per 100,000 population; CI: confidence interval; WHO: World Health Organization

Macroregion	Period	ASMR, Brazil 2022 standard (95% CI)	ASMR, WHO World Standard (95% CI)
Brazil (overall)	2000-2004	3.68 (3.63-3.73)	3.02 (2.98-3.06)
Brazil (overall)	2020-2023	1.49 (1.46-1.51)	1.18 (1.16-1.20)
North	2000-2004	0.80 (0.69-0.90)	0.65 (0.57-0.73)
North	2020-2023	0.39 (0.34-0.45)	0.31 (0.27-0.36)
Northeast	2000-2004	2.23 (2.16-2.31)	1.86 (1.80-1.92)
Northeast	2020-2023	1.42 (1.37-1.47)	1.14 (1.10-1.18)
Southeast	2000-2004	4.26 (4.19-4.34)	3.49 (3.42-3.55)
Southeast	2020-2023	1.53 (1.49-1.57)	1.22 (1.19-1.25)
South	2000-2004	1.28 (1.21-1.36)	1.05 (0.99-1.11)
South	2020-2023	0.35 (0.32-0.39)	0.28 (0.25-0.30)
Central-West	2000-2004	15.30 (14.87-15.73)	12.40 (12.06-12.75)
Central-West	2020-2023	5.18 (4.99-5.37)	4.09 (3.93-4.24)

Appendix G 

**Table 11:** Joinpoint regression of age-standardized mortality from chronic Chagas cardiomyopathy by macroregion and by sex, Brazil, 2000-2023. Same model specification and selection rules as in Table 3 (weighted log-linear joinpoint regression on the annual age-standardized rate, Bayesian information criterion, 0 to 3 joinpoints). AAPC is the length-weighted geometric mean of segment-specific APCs. APC: annual percent change; AAPC: average annual percent change; CI: confidence interval

Series	Joinpoint(s)	Segment	APC, % per year (95% CI)	P value	AAPC 2000-2023, % (95% CI)
Brazil (overall)	2020	2000-2020	−4.4 (−4.5 to −4.2)	<0.001	−4.7 (−4.9 to −4.5)
		2020-2023	−6.9 (−8.7 to −5.2)	<0.001	
North	2016	2000-2016	−0.9 (−1.9 to 0.1)	0.070	−3.7 (−4.4 to −3.0)
		2016-2023	−9.8 (−12.2 to −7.4)	<0.001	
Northeast	2008	2000-2008	0.7 (−0.3 to 1.8)	0.147	−2.2 (−2.6 to −1.9)
		2008-2023	−3.8 (−4.3 to −3.3)	<0.001	
Southeast	None	2000-2023	−5.2 (−5.4 to −4.9)	<0.001	−5.2 (−5.4 to −4.9)
South	None	2000-2023	−6.3 (−6.9 to −5.8)	<0.001	−6.3 (−6.9 to −5.8)
Central-West	2007, 2018	2000-2007	−7.5 (−8.4 to −6.5)	<0.001	−5.8 (−6.1 to −5.5)
		2007-2018	−3.1 (−3.7 to −2.6)	<0.001	
		2018-2023	−9.1 (−10.6 to −7.6)	<0.001	
Male (Brazil)	2019	2000-2019	−4.6 (−4.9 to −4.4)	<0.001	−5.0 (−5.3 to −4.8)
		2019-2023	−6.9 (−8.6 to −5.3)	<0.001	
Female (Brazil)	2020	2000-2020	−3.9 (−4.0 to −3.7)	<0.001	−4.2 (−4.4 to −4.0)
		2020-2023	−6.2 (−7.8 to −4.5)	<0.001	

Appendix H 

**Table 12:** Age-stratified trends in mortality from chronic Chagas cardiomyopathy, Brazil, 2000-2023. Rates are age-group-specific mortality rates per 100,000 population, pooled over the first (2000-2004) and last (2019-2023) five years of the series (within each broad group, the pooled rate is the sum of deaths divided by the sum of person-years, i.e., it is not standardized within the group). APC from a Poisson regression restricted to the 5-year age bands within each group, with fixed effects for age band, a linear year term, and a log-population offset; CIs scaled for overdispersion (quasi-Poisson). Deaths with unknown age were excluded. CI: confidence interval; APC: annual percent change

Age group (years)	Deaths, n	Rate 2000-2004	Rate 2019-2023	Relative change (%)	APC, % per year (95% CI)	P value
<40	3,927	0.23	0.03	−84.9	−9.9 (−10.5 to −9.3)	<0.001
40-59	24,247	4.22	1.07	−74.6	−7.3 (−7.5 to −7.1)	<0.001
60-79	44,706	14.6	6.22	−57.4	−4.2 (−4.5 to −3.9)	<0.001
≥80	16,171	22.32	20.09	−10.0	−0.5 (−0.7 to −0.2)	<0.001
